# Analysing the Implications of Charging on Nanostructured Li_2_MnO_3_ Cathode Materials for Lithium-Ion Battery Performance

**DOI:** 10.3390/ma15165687

**Published:** 2022-08-18

**Authors:** Tshidi Mogashoa, Raesibe Sylvia Ledwaba, Phuti Esrom Ngoepe

**Affiliations:** Materials Modelling Centre, University of Limpopo, Private Bag X 1106, Sovenga 0727, South Africa

**Keywords:** Li_2_MnO_3_, amorphisation, recrystallisation, nanoporous, charging, energy storage, lithium-ion battery

## Abstract

Capacity degradation and voltage fade of Li_2_MnO_3_ during cycling are the limiting factors for its practical use as a high-capacity lithium-ion battery cathode. Here, the simulated amorphisation and recrystallisation (A + R) technique is used, for generating nanoporous Li_2_MnO_3_ models of different lattice sizes (73 Å and 75 Å), under molecular dynamics (MD) simulations. Charging was carried out by removing oxygen and lithium ions, with oxygen charge compensated for, to restrain the release of oxygen, resulting in Li_2−x_MnO_3−x_ composites. Detailed analysis of these composites reveals that the models crystallised into multiple grains, with grain boundaries increasing with decreasing Li/O content, and the complex internal microstructures depicted a wealth of defects, leading to the evolution of distorted cubic spinel LiMn_2_O_4_, Li_2_MnO_3_, and LiMnO_2_ polymorphs. The X-ray diffraction (XRD) patterns for the simulated systems revealed peak broadening in comparison with calculated XRD, also, the emergence of peak 2Θ ~ 18–25° and peak 2Θ ~ 29° were associated with the spinel phase. Lithium ions diffuse better on the nanoporous 73 Å structures than on the nanoporous 75 Å structures. Particularly, the Li_1.00_MnO_2.00_ shows a high diffusion coefficient value, compared to all concentrations. This study shed insights on the structural behaviour of Li_2_MnO_3_ cathodes during the charging mechanism, involving the concurrent removal of lithium and oxygen.

## 1. Introduction

Li-ion batteries, with high energy density, are highly desired for the realisation of electric vehicles and hybrid vehicles in the automotive sectors [1,2]. Owing to its high capacity of 459 mA·g^−1^, Li_2_MnO_3_ has been considered the most promising cathode material for next-generation lithium ion batteries (LIB) [3,4]. However, activation is required before operation in its crystalline form, and this results in low charge efficiency [5]. Contrarily, in its nanocrystalline form, Li_2_MnO_3_ is instinctively active due to the presence of manganese defects, which are absent in the bulk crystalline form; these defects act as a scaffold, thus maintaining the structural integrity during the cycling process [6]. Despite its high capacity, Li_2_MnO_3_ suffers from voltage fade, irreversible capacity loss, and poor rate capability, which hinder its practical use. These poor cycling behaviours are due to irreversible oxygen loss and phase transformation, resulting from the migration of manganese ions into the lithium layers [2,7,8]. The irreversible oxygen loss is considered the crucial element behind the inadequate performance of this material [2,8]. Furthermore, oxygen released during cycling can result in gas formation, thus, put the battery at risk of expansion and performance degradation [9]. Another feature that significantly contributes to these degradations is the deformation and cracking of the electrode during cycling, resulting from stress generated due to volume expansion during cycling [10,11].

To realise structural and electrochemical improvement of the Li_2_MnO_3_ electrode, various strategies have been considered [5,12,13]. One strategy is to form a composite with the spinel LiMn_2_O_4_ [14]. The layered-spinel composite, formed by mechanical milling, displayed an initial discharge capacity of approximately 400 mA·g^−1^, which was substantially retained during the cycling process [13]. The presence of the spinel phase in the composite help restricts the emission of oxygen from the Li_2_MnO_3_, and the layered component plays a significant role in storing excess lithium [12]. Cation doping has been used to stabilize the crystal structure of Li_2_MnO_3_ and, also, reduce voltage decay [12]. Xiang et al. investigated the effect of Al doping on the Mn site of the Li_2_MnO_3_, prepared via the sol-gel method, and their results revealed that Al doping restricted structural evolution during the first charge and, also increased the rate capacity and cycling stability [15].

Another approach that has gained significant interest is the introduction of oxygen vacancies by the thermal reduction in Li_2_MnO_3_ [16]. Low-temperature reduction in Li_2_MnO_3_ nanobelts resulted in a wealth of structural defects, including oxygen vacancies and stacking faults, which contributed to the discharge capacity and lithium diffusion enhancement [5]. Furthermore, nanosizing of Li_2_MnO_3_ has been considered, due to the advantage in particle size reduction enabling large surface exposure and a shortened diffusion path, thus, facilitating effective activation of the electrode material that might reduce voltage loss and promote Li-ion migration during cycling [6,7]. Lim et al. reported that Li_2_MnO_3_ nanoparticles, prepared by oxidation reaction, exhibited excellent electrochemical properties, compared to the Li_2_MnO_3_ bulk samples [17]. Accordingly, the nanoparticles delivered a high capacity of 302 mA·g^−1^ above 4.5 V and a discharge capacity of 236 mA·g^−1^, during the first charge–discharge cycle. Despite the efforts made, there are a lot of uncertainties regarding the structural and electrochemical behaviour of the layered Li_2_MnO_3_, during the cycling process [18].

In this work, the amorphisation and recrystallisation technique is used in carrying out the simulated synthesis of nanosized porous Li_2_MnO_3_ structures, in a quest to investigate their electrochemical behaviour and structural characterisation, during the charging process. The amorphisation and recrystallisation process requires one to generate an amorphous configuration, which is, then allowed to nucleate and recrystallise under material-specific conditions. This strategy is responsible for the spontaneous growth of crystals, exhibiting microstructural features observed experimentally, such as grain boundaries, point defects, and dislocations [19]. The second aspect of significant interest is the simulated charging process, involving the systematic removal of oxygen, together with lithium. This is mainly due to the lattice oxygen loss reported previously, which results in the migration of Mn into Li layers that contributes to structural degradation during the cycling of Li_2_MnO_3_ [5]. Since the charge compensation in Li_2_MnO_3_ is maintained by the oxidation of oxygen, during which manganese ions remain in the Mn^4+^ state [20,21], the charging of the generated structures will be carried out by extracting both lithium and oxygen ions, to mitigate the initial capacity loss [7]. Finally, the diffusion coefficients will be evaluated as an electrochemical performance factor that will help diagnose the severity of oxygen loss, during the cycling process of the Li_2_MnO_3_ cathode material.

## 2. Materials and Methods

In this section, we concisely discuss the methods used for generating the Li_2_MnO_3_ atomistic models. Accordingly, the potential models that were employed in describing the interactions of the Li^+^, Mn^4+^, and O^2−^ ions, the amorphisation and recrystallisation technique, and the simulation codes responsible for the molecular dynamics simulations will, also, be discussed.

### 2.1. Potential Model and Simulation Codes 

The molecular dynamics simulations presented here are based on the Born model of ionic solids, where the components in Li_2_MnO_3_ interact, via short-range and long-range coupled coulombic interactions. The potential parameters, used for describing the Li_2_MnO_3_ interactions, were obtained from Sayle et al. [6] and are capable of reproducing the lattice parameters of pyrolusite and ramsdellite polymorphs of MnO_2_, to within 3% and 4%, respectively [6]. The DL_POLY code [22] was used to perform all the molecular dynamics simulations, and the fundamental input files (CONFIG, FIELD, CONTROL) were generated via the METADISE [23] code.

### 2.2. Generation of the Li_2_MnO_3_ Atomistic Models

The initial step was to generate an amorphous configuration, which then acted as a ‘building block’ for the desired nanoporous models. Accordingly, a nanosphere with a desired radius was cleaved from the Li_2_MnO_3_ parent bulk. To maintain the stoichiometry of the system, lithium and oxygen ions were, randomly, extracted from the outer surface of the sphere, to facilitate charge neutrality. The cleaved nanoparticle consisted of 32,148 atoms, i.e., 10,716 Li^+^ ions, 5358 Mn^4+^ ions, and 16,074 O^2−^ ions. The nanoparticle (9.73 nm) was then placed at the centre of a simulation cell, with dimensions sufficiently high (in all directions) to prohibit neighbouring nanoparticles from, first, attracting one another and, then, agglomerating upon contact. An amorphous configuration was achieved by heating the spherical system to 1850 K [6], within the NVE (constant number of atoms, constant volume, and constant energy) ensemble, as this temperature was sufficient to overcome the forces of attraction within the Li_2_MnO_3_ system.

The amorphous nanoparticle cell size was reduced, in all three dimensions (3D), to enable the nanoparticles to agglomerate in all three spatial directions, to facilitate the formation of nanoporous structures of different cell sizes (7.30 and 7.50 nm), as previously attained for MnO_2_ and LiMn_2_O_4_ electrodes [11,19,24]. These were carried out for 6 ns, with time steps set to 0.001 ps and Ewald precision of 5, while the Nosé–Hoover thermostat was used to maintain the 1850 K temperature and 50 Gpa under the NPT (constant number of atoms, constant pressure and constant temperature) ensemble. These conditions were sufficient to assist with the inter-nanocrystal attraction for ultimate agglomeration, within the time scale accessible to the MD simulations.

**Figure 1:**
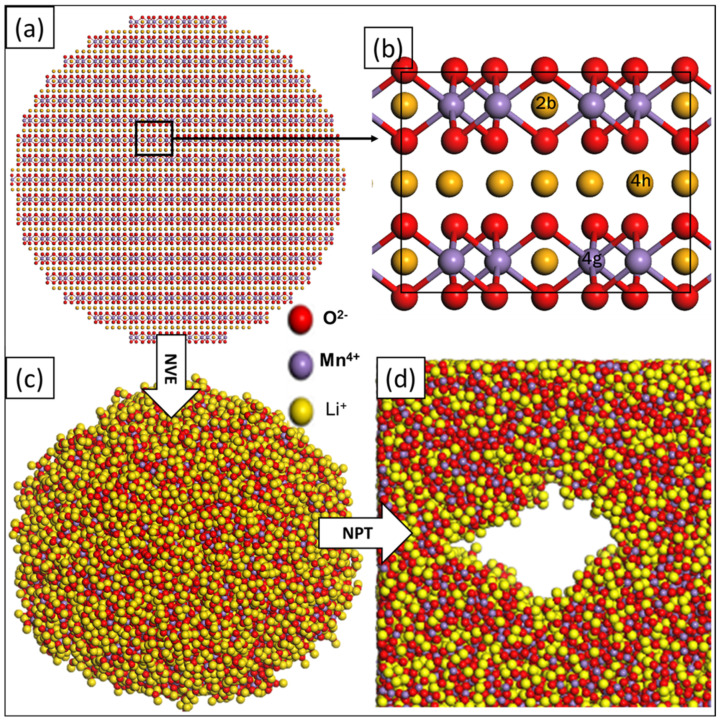
Schematic diagrams representing (**a**) the spherical layered Li_2_MnO_3_, (**b**) magnified portion of (**a**) revealing the atomic arrangement of Li, Mn, and O, (**c**) amorphous nanosphere Li_2_MnO_3_, and (**d**) amorphous nanoporous Li_2_MnO_3_ configurations.

To recrystallise the models, molecular dynamics (MD) simulations were performed at 1850 K for 6 ns, using the NVT (constant number of atoms, constant volume, and constant temperature) ensemble. In an attempt to achieve low-energy models, the systems were allowed to cool, systematically, by gradually decreasing the temperature from 1850 K to 5 K. A schematic illustration of the Li_2_MnO_3_ synthesis procedure is presented in Figure 1 indicating (a) the spherical Li_2_MnO_3_ with 32,148 atoms cleaved from the parent bulk; a portion is magnified in (b), revealing the atomic arrangement conforming to a monoclinic structure with C2/m symmetry, while (c) is the amorphous precursor used as the building blocks for (d), nanoporous architectures. 

In order to mimic the charging process, the simulated synthesis of Li_2−x_MnO_3−x_ (x = 0.25, 0.50, 0.75 and 1.0) systems was carried out, by, systematically, removing the same concentration of lithium and oxygen ions, simultaneously, from the outer surface of the amorphous nanoparticle and compensating the oxygen charge. This facilitated four systems with different lithium and oxygen contents, i.e., Li_1.75_MnO_2.75_, Li_1.50_MnO_2.50_, Li_1.25_MnO_2.25_, and Li_1.00_MnO_2.00_.

## 3. Results and Discussions

### 3.1. Radial Distribution Functions (RDFs)

**Figure 2:**
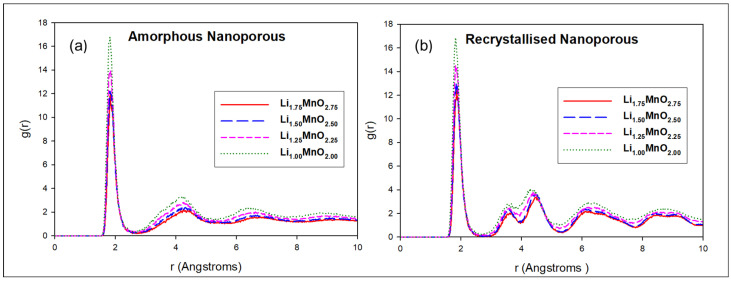
Mn–O pair distribution for the nanoporous 75 Å, before (**a**) and after (**b**) recrystallisation, where Li_1.75_MnO_2.75_, Li_1.50_MnO_2.50_, Li_1.25_MnO_2.25_, and Li_1.00_MnO_2.00_ concentrations are illustrated by red, blue, pink, and green, respectively.

The RDFs associated with the Mn–O interactions were plotted to confirm the amorphisation and recrystallisation of the generated nanoporous Li_2−x_MnO_3−x_ structures, as their Li and O content was varied. Figure 2a illustrates the amorphous plots for the Li_1.75_MnO_2.75_ (red), Li_1.50_MnO_2.50_ (blue), Li_1.25_MnO_2.25_ (pink), and Li_1.00_MnO_2.00_ (green) structures, revealing a sharp peak at 1.9 Å corresponding to the Mn–O bond length, which is comparative to the 1.92 Å in the literature [25]. The peaks beyond 5 Å are broader, with minimal possibility of locating the nearest neighbouring atom within the radial distance, thus confirming that the structures were amorphised.

In Figure 2b, the recrystallised plots for the Li_1.75_MnO_2.75_ (red), Li_1.50_MnO_2.50_ (blue), Li_1.25_MnO_2.25_ (pink), and Li_1.00_MnO_2.00_ (green) structures show an increased number of sharp peaks, due to strong bonds and a high probability of locating the nearest neighbouring atom, within the radial distance. It can be deduced from the graphs that the Li_1.00_MnO_2.00_ structure is highly crystalline, since it recorded the highest g (r) value, compared to the other structures. The presence of multiple sharp peaks confirms that the models are in their crystalline form.

### 3.2. Structural Analysis

**Figure 3:**
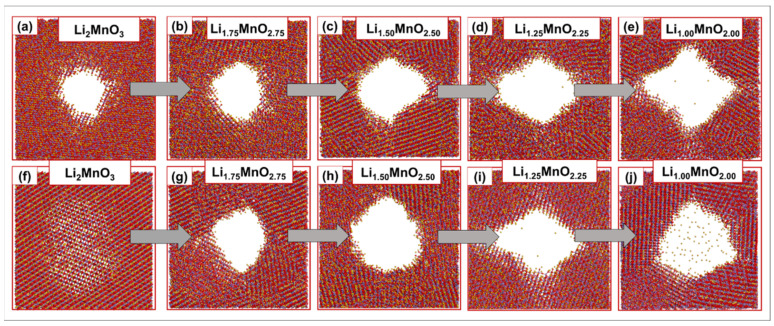
Molecular graphics of the crystallised Li_2−x_MnO_3−x_ (**a**–**e**) nanoporous 75 Å and (**f**–**j**) nanoporous 73 Å, with different Li/O concentrations.

Figure 3 illustrates molecular graphics for the recrystallised nanoporous 75 Å (a–e) and nanoporous 73 Å (f–j) Li_2−x_MnO_3−x_ models, with different Li/O content. The observable patterns, within the simulated models, are an indication of their crystallinity. As the Li/O content was varied, the pore size gradually increased, along with the grain boundaries, for the nanoporous 75 Å (a–e). Moreover, the pristine Li_2_MnO_3_ pore depicted in (f) closes on one side upon crystallisation, opening up as the Li/O content decreased for the nanoporous 73 Å. Moreover, at the same lattice size, there are considerable lithium ions located within the LiMnO_2_ pore, shown in (j), which shows that the removal of Li/O affects the pore size of the generated Li_2−x_MnO_3−x_ models.

**Figure 4:**
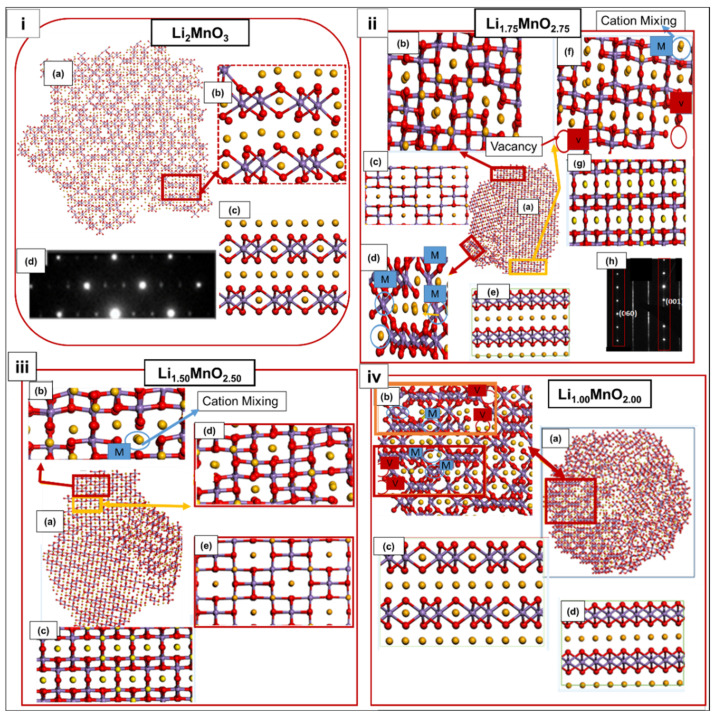
[**i**] (**a**) Cut-through Li_2_MnO_3_, (**b**) magnified portion from (**a**) depicting the Li_2_MnO_3_ component, (**c**) Li_2_MnO_3_ perfect model and (**d**) Li_2_MnO_3_ SAED experimental patterns reproduced with permission from Ref. [5]. 2017. Advanced Materials Interfaces. [**ii**] (**a**) Cut-through Li_1.75_MnO_2.75_, (**b**) magnified segment from (**a**) illustrating the distorted spinel LiMn_2_O_4_ phase, (**c**) LiMn_2_O_4_ perfect model, (**d**) magnified portion from (**a**) showing the distorted LiMnO_2_ component, comparable with (**e**) the LiMnO_2_ perfect model, (**f**) magnified portion from (**a**) revealing the Li_2_MnO_3_ component, (**g**) perfect Li_2_MnO_3_ model, and (**h**) Li_2_MnO_3_ SAED reproduced with permission from Ref. [26]. 2015. Nano Energy. [**iii**] (**a**) Cut-through Li_1.50_ MnO_2.50_, revealing (**b**) Li_2_MnO_3_ component, (**c**) Li_2_MnO_3_ perfect model, (**d**) magnified portion from (a) showing the LiMn_2_O_4_ component, comparable with (**e**) the LiMn_2_O_4_ perfect model. [**iv**] (**a**) Cut-through Li_1.00_MnO_2.00_ depicting (**b**) magnified portion of (**a**) revealing the coexistence of the layered Li_2_MnO_3_ and LiMnO_2_ components, comparable with (**c**) the Li_2_MnO_3_ perfect model and (**d**) LiMn_2_O_4_ perfect model.

To explore the crystallographic features that evolved during the charging of the nanoporous structures, the structures were cut through into segments, to better view their atomic arrangement. Figure 4 illustrates the cut-through nanoporous 75, with Figure 4(ia) pristine Li_2_MnO_3_ showing cation mixing, as indicated on the magnified portion (b); the structure compares well with the Li_2_MnO_3_ perfect model in (c) and, also, with the experimental Li_2_MnO_3_ selected area electron diffraction (SAED) [5], depicted in (d). In Figure 4(iia), the cut-through Li_1.75_MnO_1.75_ has evolved into three disoriented phases, the spinel LiMn_2_O_4_ phase, magnified in (b) and compared with the spinel LiMn_2_O_4_ perfect model in (c); the LiMnO_2_ phase magnified in (d) accompanied by cation mixing and vacancies, compared with the LiMnO_2_ perfect model in (e); the Li_2_MnO_3_ phase magnified in (f) also, showing cation mixing and vacancies compared with the Li_2_MnO_3_ perfect model in (g) and Li_2_MnO_3_ SAED [26] in (h). Figure 4(iiia) depicts a cut-through Li_1.50_MnO_2.50_, comprising the layered Li_2_MnO_3_ phase indicated in (b), comparable to the Li_2_MnO_3_ perfect model in (c) and also, the spinel LiMn_2_O_4_ phase, comparable to (e), the LiMn_2_O_4_ perfect model. In Figure 4(iva), the cut-through Li_1.00_MnO_2.00_, also, reveals the coexistence of the layered Li_2_MnO_3_ and LiMnO_2_ phases magnified in (b), which are, also, comparable to their perfect models depicted in (c) Li_2_MnO_3_ and (d) LiMnO_2_.

**Figure 5:**
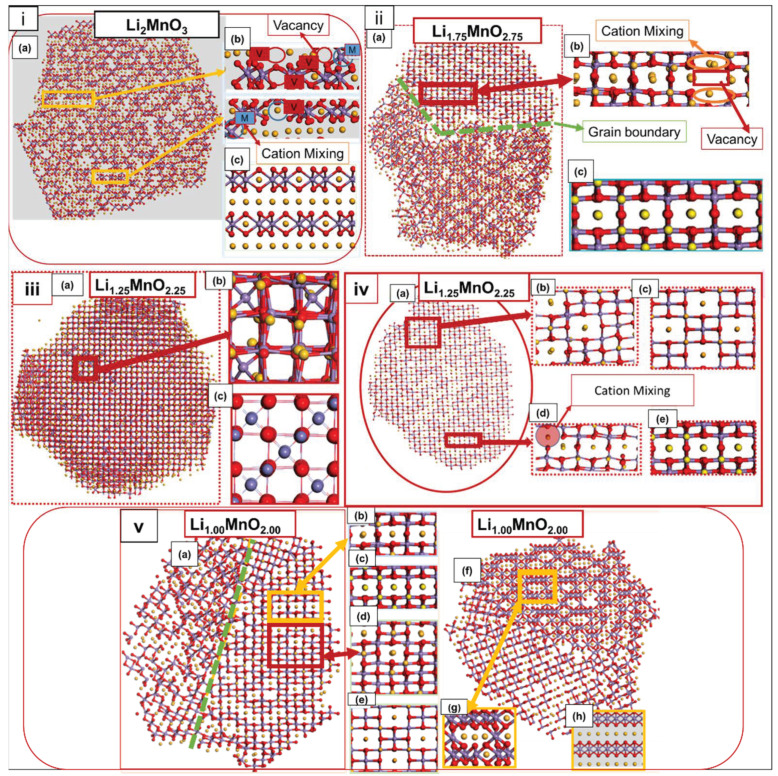
[**i**]. (**a**) A cut-through Li_2_MnO_3_ and (**b**) magnified portion of the cut through, illustrating the Li_2_MnO_3_ component, comparable with (**c**) the cut-through Li_2_MnO_3_ perfect model. [**ii**] (**a**) Cut-through Li_1.75_MnO_2.75_, (**b**) magnified portion of (**a**) revealing the Li_2_MnO_3_ component, comparable with (**c**) the perfect Li_2_MnO_3_ model. [**iii**] (**a**) Li_1.25_MnO_2.25_ atomic structures, revealing (**b**) spinel Mn_3_O_4_ component, comparable with (**c**) the Mn_3_O_4_ perfect model. [**iv**] (**a**) Cut-through Li_1.25_MnO_2.25_, depicting (**b**) spinel LiMn_2_O_4_ component, comparable with (**c**) LiMn_2_O_4_ perfect model, (**d**) magnified portion from (**a**) illustrating the Li_2_MnO_3_ component, comparable with (**e**) Li_2_MnO_3_ perfect model. [**v**] (**a**) Cut-through Li_1.00_MnO_2.00_ revealing the (**b**) Li_2_MnO_3_ component, comparable with (**c**) Li_2_MnO_3_ perfect model, (**d**) magnified segment from (**a**) illustrating the LiMn_2_O_4_ component, comparable with (**e**) LiMn_2_O_4_ perfect model, (**f**) cut through Li_1.00_MnO_2.00_ viewed from different orientation revealing (**g**) magnified portion of (**f**) showing, LiMnO_2,_ component, comparable with (**h**) LiMnO_2_ perfect model.

Figure 5 represents the nanoporous 73 Å microstructures, respectively. A cut-through Li_2_MnO_3_ is depicted in Figure 5(ia), while a magnified portion (b) reveals cation mixing (blue circle) and vacancies (red circle), within the layers of Li_2_MnO_3_, (c) is the Li_2_MnO_3_ perfect model. Similarly, the Li_1.75_MnO_2.75_ cut through, presented in Figure 5ii, reveals (a) the distorted Li_2_MnO_3_, along with the grain boundaries specified by the green dotted line. Figure 5iii indicates (a) the Li_1.25_MnO_2.25_ snapshot, magnified in (b) to better view the occupancy of Mn ions in the tetrahedral sites arranged, correspondingly, as in (c) the spinel Mn_3_O_4_ perfect model. Moreover, in Figure 5iv, the cut-through Li_1.25_MnO_2.25_ is displayed in (a) and magnified in (b), to show the cubic-spinel phase LiMn_2_O_4_, comparable with (c) the LiMn_2_O_4_ perfect model, while another magnified portion (d) reveals the layered Li_2_MnO_3_, showing Li-ion substituting for the Mn ion (red circle), comparable with (e) the Li_2_MnO_3_ perfect model. The Li_1.00_MnO_2.00_ microstructural features are depicted in Figure 5iv, with (a,f) illustrating the cut-through Li_1.00_MnO_2.00_ layers, viewed in a different orientation for better analysis. The magnified portions (b) indicate the presence of the Li_2_MnO_3_ phase, comparable to (c) the Li_2_MnO_3_ perfect model, while (d) shows the presence of LiMn_2_O_4_, comparable to (e) the perfect LiMn_2_O_4_, and (g) depicts the presence of the LiMnO_2_ phase, which is, also, comparable to (h) the LiMnO_2_ perfect model. Our results reveal that the pristine models of Li_2_MnO_3_ recrystallised into single crystals with a distorted rocksalt structure, similar to that of the nanoparticle simulated in a previous study [6]. As the Li/O content was decreased, the structures crystallised into multiple grains along with stacking faults and vacancies, thus leading to Mn ions migrating to the Li layers.

Our models reveal that all layers are mixed upon crystallisation, leading to the formation of disoriented phases, including the layered Li_2_MnO_3_, layered LiMnO_2_, and cubic-spinel LiMn_2_O_4_. Furthermore, the models showed the presence of grains, which increased along with Li/O extraction, however, for nanoporous 73 Å, the Li_1.25_MnO_2.25_ concentration depicted no grain boundaries. The resulting structures crystallised into multiple grains, which increased with decreasing Li/O content, along with stacking faults and vacancies, thus leading to Mn ions migrating to the Li layers. Interrogating the structural composition of the charged structures revealed the presence of spinel-layered-type polymorphs, consisting of a 3D spinel framework and straight tunnels (layered structure), which would allow for efficient lithium-ion diffusion during intercalation, as described in the literature for the parent bulk structures [7]. The polymorphs that emanated during the charging process were compared with the SAED images [5,26] from experimental studies. The spinel components contain Li atoms that, predominantly, reside in the octahedral 16c and 16d sites, compared to the tetrahedral 8a positions of pure LiMn_2_O_4_. Another crucial component, which emanated from lower oxygen and lithium contents, was the LiMnO_2_ polymorph. This, also, affects the precise structural analysis, due to the level of defects formed within the crystal domains [27].

### 3.3. X-ray Diffraction Patterns (XRDs)

For further characterisation, XRDs for the calculated nanoporous Li_2−x_MnO_3−x_ models were compared with the ones from the literature, to consider whether structural features corresponding to those of known metal oxides may have evolved during the recrystallisation and charging process.

**Figure 6:**
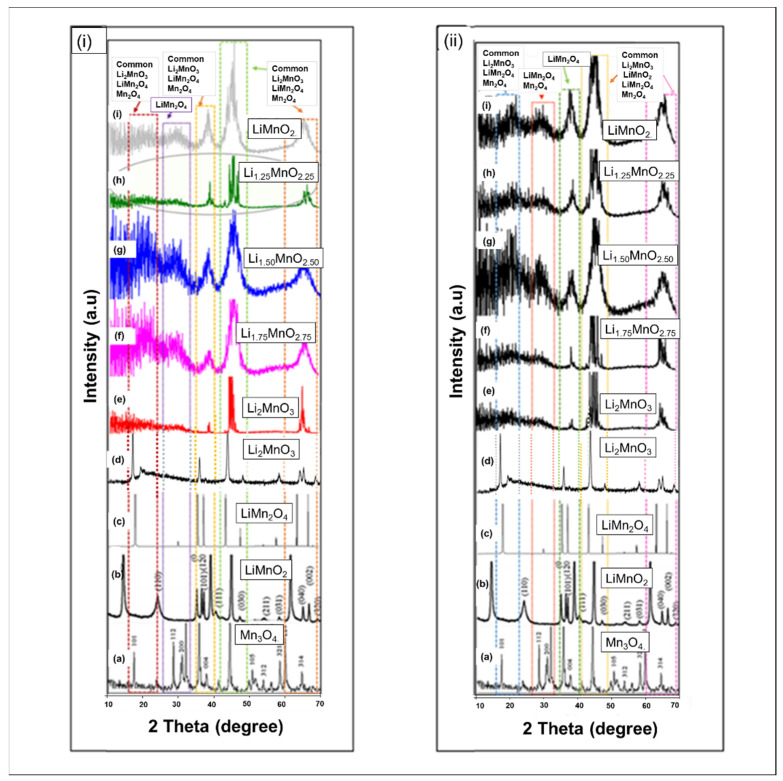
[**i**] XRDs graphs for the (**a**) Mn_3_O_4_ reproduced with permission from Ref. [28]. 2017. Water Conservation Science and Engineering, (**b**) LiMnO_2_ reprodused with permission from Ref. [29]. 2008. Journal of Solid State Electrochemistry, (**c**) LiMn_2_O_4_ [29], (**d**) Li_2_MnO_3_ reproduced with permission from Ref. [15]. 2018. Ionics., superimposed with simulated XRDs calculated from the nanoporous 73 Å, (**e**) Li_2_MnO_3_, (**f**) Li_1.75_MnO_2.75_, (**g**) Li_1.50_ MnO _2.50_, (**h**) Li_1.25_ MnO_2.25_, and (**i**) LiMnO_2_. [**ii**] diffraction peaks for the the (**a**) Mn_3_O_4_ [28], (**b**) LiMnO_2_, (**c**) LiMn_2_O_4_ [29], (**d**)Li_2_MnO_3_ [15], superimposed with simulated XRDs calculated from nanoporous 75 Å, (**e**) Li_2_MnO_3_, (**f**) Li_1.75_MnO_2.75_, (**g**) Li_1.50_ MnO _2.50_, (**h**) Li_1.25_ MnO_2.25_, and (**i**) LiMnO_2_.

Figure 6 illustrates the XRD patterns for the (i) nanoporous 73 Å and (ii) nanoporous 75 Å, during the charging process. The XRDs of experimentally synthesized (a) MnO_3_ [28], (b) LiMnO_2_, (c) LiMn_2_O_4_ [29] and (d) Li_2_MnO_3_ [15] are superimposed with the calculated XRD patterns for the simulated systems (e-i). The simulated XRDs display a broad character at lower angles, corresponding to the ordering of atoms in the transition metal layers, thus resulting in highly distorted structures, as observed on the microstructures [8].

In Figure 6i, a shoulder peak associated with the Li_2_MnO_3_, Mn_3_O_4_, and LiMn_2_O_4_ phase emerges at lower angles (2Θ ~ 18–25°), while another peak associated with Mn_3_O_4_ emerges at 2Θ ~ 29°. These peaks drastically decrease for the Li_1.25_MnO_2.25_ concentration and become narrowed. The same is observed at 2Θ ~ 37°, 2Θ ~ 47°, and 2Θ ~ 68°, where the peaks increase but decrease for the Li_1.25_MnO_2.25_ concentration. This concentration has, also, revealed fewer grain boundaries during recrystallisation. Figure 6ii, also, reveals the emergence of shoulder peaks at low angles, and the peak at 2Θ ~ 37° increases with a decrease in Li/O content. The multiple peaks at 2Θ ~ 47° and 2Θ ~ 68° merge into a broader peak that shifts to the right, as the Li/O content decreases, while the peaks correspond to the spinel phase. At lower concentrations (Li_1.00_MnO_2.00_), the diffraction peaks are broader, as a result of an increased grain boundary, as seen on the microstructures.

The XRD of the simulated systems depicts broader peaks, which are more intense at lower angles, corresponding to the ordering of atoms in TM layers, denoted as the superionic feature in Li_2_MnO_3_. The spinel and layered morphologies observed from the microstructures are, also, observed in the XRD analysis. Again, the nanoporous 73 Å Li_1.25_MnO_2.25_ XRDs is narrowed, compared to the other concentrations. A drastic decrease is observed from the flattening of peaks, for both spinel and layered components. This concentration has, also, revealed fewer grain boundaries during recrystallisation. As previously reported by Xiao et al., the presence of high contents of Mn^3+^ highly enhances the activation of Li_2_MnO_3_ and, thus, the electrochemical performance [26].

### 3.4. Diffusion Coefficient

**Figure 7:**
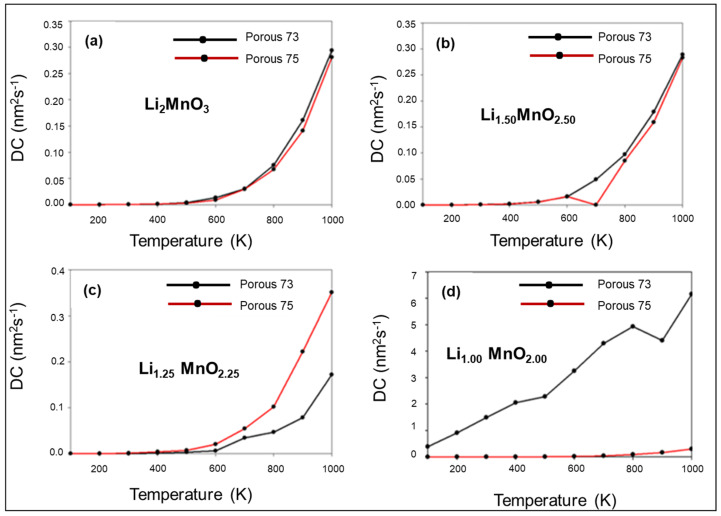
Diffusion coefficient plots for Li-ions in (**a**) Li_2_MnO_3_, (**b**) Li_1.50_MnO_2.50_, (**c**) Li_1.25_MnO_2.25_, and (**d**) Li_1.00_MnO_2.00_, at various temperatures, where black trace represents nanoporous 73 Å and red trace represents nanoporous 75 Å, respectively.

Li-ion diffusion was investigated at different temperatures and charge states (Li_2−x_MnO_3−x_). Figure 7a illustrates the diffusion coefficient (DC) of the Li_2_MnO_3_ nanoporous 75 Å (red trace) and nanoporous 73 Å (black trace). Li-ion in nanoporous 73 Å diffuses slightly higher than the nanoporous 75 Å, while Li mobility is observed at 500 K, and the diffusion coefficient increases, exponentially, until 1000 K. The same pattern is observed in Figure 7b, as both the Li_1.50_MnO_2.50_ porous structures show Li motion at 500 K; however, for the nanoporous 75 Å, Li diffusion drops to zero at 700 K and increases, again, until 1000 K.

In Figure 7c, the Li_1.25_MnO_2.25_ systems also depict Li mobility starting at 500 K, with nanoporous 75 Å showing better Li diffusivity than porous 73 Å. In terms of the Li_1.00_MnO_2.00_ structures (Figure 7d), Li mobility is observed at a lower temperature (100 K) for the nanoporous 73 Å and significantly increases until 800 K, before it slightly drops and increases, again, until 1000 K. This sudden change may be attributed to severe structural transformation, emanating from the removal of Li/O content.

## 4. Conclusions

We generated Li_2−x_MnO_3−x_ nanoporous structures of different cell sizes (73, 75 Å) and interrogated their microstructures, XRDs, and lithium-ion diffusion, during the charging involving the simultaneous removal of oxygen and lithium ions. Our results display highly distorted structures, emanating from factors including stacking faults, vacancies, cation-mixing and splitting, and displacement of the XRD patterns. The presence of the layered (Li_2_MnO_3_, LiMnO_2_) and spinel (Mn_3_O_4_, LiMn_2_O_4_) polymorphs was deduced from the microstructures and XRDs. Lithium-ion diffusion was more favorable on the nanoporous 73 Å, particularly for Li_1.00_MnO_2.00_, which showed the highest diffusion coefficient value. This study has revealed the different internal structural transitions that occur within the intermediate phases and their impact on the cycling capabilities of Li_2_MnO_3_ cathodes, while similar results were observed, experimentally [2,13].

## Data Availability

The data presented in this work are available on request from the corresponding author.
